# The Predictive Performance of a Pneumonia Severity Score in Human Immunodeficiency Virus-negative Children Presenting to Hospital in 7 Low- and Middle-income Countries

**DOI:** 10.1093/cid/ciz350

**Published:** 2020-03-03

**Authors:** Katherine E. Gallagher, Maria D. Knoll, Chrissy Prosperi, Henry C. Baggett, W. Abdullah Brooks, Daniel R. Feikin, Laura L. Hammitt, Stephen R. C. Howie, Karen L. Kotloff, Orin S. Levine, Shabir A. Madhi, David R. Murdoch, Katherine L. O’Brien, Donald M. Thea, Juliet O. Awori, Vicky L. Baillie, Bernard E. Ebruke, Doli Goswami, Alice Kamau, Susan A. Maloney, David P. Moore, Lawrence Mwananyanda, Emmanuel O. Olutunde, Phil Seidenberg, Seydou Sissoko, Mamadou Sylla, Somsak Thamthitiwat, Khalequ Zaman, J.Anthony G. Scott, for the Pneumonia Etiology Research for Child Health (PERCH) Study Group

**Affiliations:** 1 Department of Infectious Disease Epidemiology, Faculty of Epidemiology and Population Health, London School of Hygiene and Tropical Medicine, United Kingdom; 2 Department of International Health, International Vaccine Access Center, Johns Hopkins Bloomberg School of Public Health, Baltimore, Maryland; 3 Division of Global Health Protection, Center for Global Health, Centers for Disease Control and Prevention (CDC), Atlanta, Georgia; 4 Global Disease Detection Center, Thailand Ministry of Public Health —US CDC Collaboration, Nonthaburi; 5 Department of International Health, Johns Hopkins Bloomberg School of Public Health, Baltimore, Maryland; 6 International Centre for Diarrhoeal Disease Research, Bangladesh, Dhaka and Matlab; 7 Division of Viral Diseases, National Center for Immunization and Respiratory Diseases, CDC, Atlanta, Georgia; 8 Kenya Medical Research Institute-Wellcome Trust Research Programme, Kilifi; 9 Medical Research Council Unit, Basse, The Gambia; 10 Department of Paediatrics, University of Auckland, New Zealand; 11 Centre for International Health, University of Otago, Dunedin, New Zealand; 12 Division of Infectious Disease and Tropical Pediatrics, Department of Pediatrics, Center for Vaccine Development, University of Maryland School of Medicine, Baltimore; 13 Medical Research Council, Respiratory and Meningeal Pathogens Research Unit; 14 Department of Science and Technology/National Research Foundation, Vaccine Preventable Diseases Unit, University of the Witwatersrand, Johannesburg, South Africa; 15 Department of Pathology, University of Otago; 16 Microbiology Unit, Canterbury Health Laboratories, Christchurch, New Zealand; 17 Department of Global Health, Boston University School of Public Health, Massachusetts; 18 Division of Global HIV and TB, Center for Global Health, CDC, Atlanta, Georgia; 19 Department of Paediatrics and Child Health, Chris Hani Baragwanath Academic Hospital and University of the Witwatersrand, Johannesburg, South Africa; 20 Center for Global Health and Development, Boston University School of Public Health, Massachusetts; 21 EQUIP-Zambia, Lusaka; 22 Centre pour le Développement des Vaccins, Bamako, Mali

**Keywords:** prognosis/prognostic scores, severity index, pneumococcal disease, respiratory disease, pneumonia

## Abstract

**Background:**

In 2015, pneumonia remained the leading cause of mortality in children aged 1−59 months.

**Methods:**

Data from 1802 human immunodeficiency virus (HIV)−negative children aged 1–59 months enrolled in the Pneumonia Etiology Research for Child Health (PERCH) study with severe or very severe pneumonia during 2011−2014 were used to build a parsimonious multivariable model predicting mortality using backwards stepwise logistic regression. The PERCH severity score, derived from model coefficients, was validated on a second, temporally discrete dataset of a further 1819 cases and compared to other available scores using the C statistic.

**Results:**

Predictors of mortality, across 7 low- and middle-income countries, were age <1 year, female sex, ≥3 days of illness prior to presentation to hospital, low weight for height, unresponsiveness, deep breathing, hypoxemia, grunting, and the absence of cough. The model discriminated well between those who died and those who survived (C statistic = 0.84), but the predictive capacity of the PERCH 5-stratum score derived from the coefficients was moderate (C statistic = 0.76). The performance of the Respiratory Index of Severity in Children score was similar (C statistic = 0.76). The number of World Health Organization (WHO) danger signs demonstrated the highest discrimination (C statistic = 0.82; 1.5% died if no danger signs, 10% if 1 danger sign, and 33% if ≥2 danger signs).

**Conclusions:**

The PERCH severity score could be used to interpret geographic variations in pneumonia mortality and etiology. The number of WHO danger signs on presentation to hospital could be the most useful of the currently available tools to aid clinical management of pneumonia.

In 2015, pneumonia remained the leading cause of mortality in children between 1 month and 5 years of age, causing 12.8% of all deaths [1]. The etiology of an individual’s pneumonia is often difficult to ascertain, and clinical signs and symptoms are the only information available to inform treatment [2–5].

In 2013, the World Health Organization (WHO) recommended the reclassification of pneumonia cases into just 2 strata to inform treatment in resource-limited settings (Supplementary Table 1) [5, 6]. This dichotomous classification does not allow us to control well for severity in pneumonia etiology studies across different geographical settings or to inform treatment. A number of scoring systems have been developed that attempt to further stratify cases by severity. However, these use data from single African sites with unknown generalizability [4, 7, 8]; the Respiratory Index of Severity in Children (RISC) was derived from South African clinical trial participants aged 1–23 months hospitalized for respiratory tract infection [4]. Additionally, WHO’s Pocketbook of Hospital Care for Children identifies common danger signs in hospitalized children that may indicate severity of disease and deterioration of condition.

To fulfill an original objective of the Pneumonia Etiology Research for Child Health (PERCH) study [9], we used data collected prospectively in 2011–2014 in 7 low- and middleincome countries (LMICs), to develop and validate a severity score (“PERCH score”) to standardize the definition of pneumonia case severity for etiology analyses. To assess whether this score might aid clinical management of pneumonia cases, we compared the performance of this PERCH score with other available scores and classifications.

## Methods

### Study Population

The PERCH study aimed to determine the etiology of pneumonia in children aged 1–59 months presenting to hospital [9]. Cases were eligible for enrollment if they were admitted to a study hospital with severe or very severe pneumonia, according to the 2005 WHO definitions (Supplementary Table 1), at 9 centers in Kenya, Zambia, South Africa, Mali, The Gambia, Bangladesh, and Thailand [10]. Standardized questionnaires and diagnostics were used across all sites [11, 12]. Clinical data were obtained at the time of admission to hospital, and mortality status was obtained in hospital or 30 days after discharge.

Of the 4232 pneumonia cases enrolled into the PERCH study, data from those who tested HIV negative (n = 3596) and those without an HIV test result but presumed negative based on parental report (n = 366) were used for model development and validation. Participants missing mortality data (8.6%) were similar in baseline characteristics to those with mortality data, but predominantly came from 1 site (Zambia; Supplementary Table 8).

Data from participants with information on the outcome (n = 3621) were split in half; the first half (enrolled August 2011−mid-November 2012; n = 1802) was used to develop the model, and the second half (enrolled mid-November 2012– January 2014; n = 1819) was used to validate the model. A temporal split allowed some non-random variation between the datasets and provided a stronger design for the validation of the model [13].

A total of 1802 HIV-negative cases in the development dataset and 120 deaths allowed us to produce a robust model containing ≤12 variables [14, 15].

### Model Development

The primary outcome of “death in hospital or within 7 days of discharge,” regardless of the duration of admission, included children who died during admission or after discharge home for convalescent care or to a higher-level facility. From the list of PERCH variables [11], potentially predictive covariates for death and/or pneumonia severity were identified, informed by previous literature [11, 16, 17], including sociodemographic characteristics, medical history, anthropometrics, the results of clinical examination and diagnostic tests, and environmental exposures. Variables with >25% missing data (eg, medication history, gestational age, weak pulse) were excluded from the analysis. A total of 54 variables were assessed in univariable analyses; “country site” was used as a forced variable to identify the strongest predictors of the outcome, controlling for clustering.

Covariates associated with mortality in univariable analyses with *P* < .2 were included in multivariable regression models. If multiple potentially collinear variables were strongly associated with the outcome (eg, the anthropometric variables), then clinical insight was used to choose the most practical variable to include [18]. The number of variables in the model was reduced using backwards stepwise logistic regression. Interactions between all the variables in the final model were assessed. Variables and interaction terms were kept in the model if they significantly improved the fit of the model (likelihood ratio test [LRT], *P* < .05). Each excluded variable was re-added to the final model to ensure the final model fit the data best.

### Evaluation of Model Performance

The forced variable of “site” was removed from the model to evaluate the performance of the model in the absence of information on clustering, as this is unlikely to be available in clinical settings. The ability of the model to discriminate between those who died and who survived was assessed using the C statistic, equivalent to the area under the receiver operating characteristic curve [19]. The C statistic ranges from 0.5 to 1; 0.5 indicates that the model is unable to discriminate beyond random chance [20]. A C statistic >0.8 was a priori defined as a good fit of the model to the data; >0.7 was defined as a moderate fit.

### PERCH Score Development and Temporal Validation

Each covariate’s coefficient on the log scale in the final model, excluding “site,” was rounded to the nearest 0.5 and then doubled to form an integer [4, 21, 22]. Five strata of severity, the “5-stratum score,” were developed by splitting the score into quintiles of roughly equal size.

The PERCH score was then computed for each record with complete data on all the score variables in the validation dataset. The ability of the 5-stratum score to discriminate between those who died and those who survived was assessed using the C statistic; bootstrapping with 200 repetitions was used to adjust for optimism [19, 21, 23]. The model’s calibration was assessed by visually comparing the predicted risk of death using the logistic regression model, with the observed deaths.

Cases in the validation dataset were also categorized according to 3 other severity classifications: (1) WHO 2005 definitions of severe pneumonia or very severe pneumonia [24]; (2) the South Africa RISC score [4]; and (3) the number of danger signs from the WHO Pocketbook of Hospital Care for Children [24]. The RISC score for HIV-negative children [4] assigns scores for ≤90% oxygen saturation on pulse oximetry, lower chest wall indrawing, low weight for age, refusal to feed, and wheeze (Supplementary Table 2). Danger signs included central cyanosis or oxygen saturation <90% on pulse oximetry, inability to drink/feed, vomiting, convulsions, lethargy (or impaired consciousness), and severe respiratory distress [24]. The C statistics of the WHO 2005 classification, the RISC score, the number of danger signs, and the PERCH 5-stratum score were compared to determine the score most able to discriminate between those who died and those who survived. The calibration between predicted deaths and observed deaths was assessed visually. The positive and negative predictive values of each of the scores were calculated by dividing the number of observed deaths by the number of predicted deaths. For this purpose, a predicted death was defined as a case with a predicted probability of death of ≥0.2, as no cases had a predicted probability of death ≥0.5.

The PERCH study was approved by the relevant ethics committee(s) overseeing each site and by the Johns Hopkins Bloomberg School of Public Health ethics committee. Written informed consent was obtained from parents/guardians prior to any study procedures.

## Results

Among the 1994 HIV-negative children in the development dataset, 1802 (90.4%) had data on survival up to 7 days post-discharge. The median age at presentation to hospital was 9 months (interquartile range [IQR], 4–19 months), 57% were male, the median duration of hospitalization was 4 days (IQR, 3–7 days), and 120 (6.7%) died within 7 days of discharge. Most of the deaths (67/120 [56%]) occurred within 48 hours of admission; 46 died later during admission (range, 3-49 days), and 7 died within 7 days of being discharged home in a moribund state or to a higher-level facility. Mortality varied by country, from 1.5% in Bangladesh to 26.0% in Zambia (Table 1). Factors associated with mortality among HIV-negative children aged 1–59 months presenting to hospital with severe or very severe pneumonia are shown in Supplementary Table 3. Presentation with at least 1 symptom of central cyanosis, hypoxemia, inability to feed, convulsions, or unresponsiveness was associated with 4.8 times higher odds of death compared to those with no symptoms, controlling for site (95% confidence interval, 3.02-7.63; Supplementary Table 3).

### The Multivariable Predictive Model

Predictors of mortality in multivariable analyses, controlling for country, were younger age, female sex, >2 days of illness prior to presentation to hospital, low weight for height, unresponsiveness, deep breathing, hypoxemia, and grunting. Observed cough was predictive of survival (Table 1). Overall, mortality was slightly lower among the 1719 observations (95%) in the final model (6.2%) than in the overall population. The predictive performance of *the model* as measured by the C statistic was good (C statistic = 0.86).

Assigned scores ranged from –1 to 16 (mean, 4.3 [standard deviation, 2.9]) (Table 2). The 5-stratum score performed well during internal validation on the development dataset (C statistic = 0.82; Supplementary Figure 1). There was good calibration between the observed and predicted prevalence of mortality. In the highest stratum, the observed mortality was 21%. The final model was rerun as a multilevel model with random effects to control for clustering. The point estimates were not substantially different from those generated using fixed effects. Fixed effects were used in the final model to allow for site-specific analyses.

### Performance of PERCH Score on the Validation Dataset

Children in the validation dataset, who were HIV negative and had data on the outcome (n = 1819), were slightly younger than in the development dataset (median age, 7 vs 9 months) and mortality was higher (8% vs 6.7%) (Supplementary Table 4). A total of 1755 of 1819 (96%) records in the validation dataset had data on all the variables included in the multivariable model. The predictive performance of the model in the validation dataset was good (C statistic = 0.84). The mean score allocated was similar to the development dataset (mean, 4.4 [standard deviation, 2.8]; range, –1 to 16).

**Figure 1:**
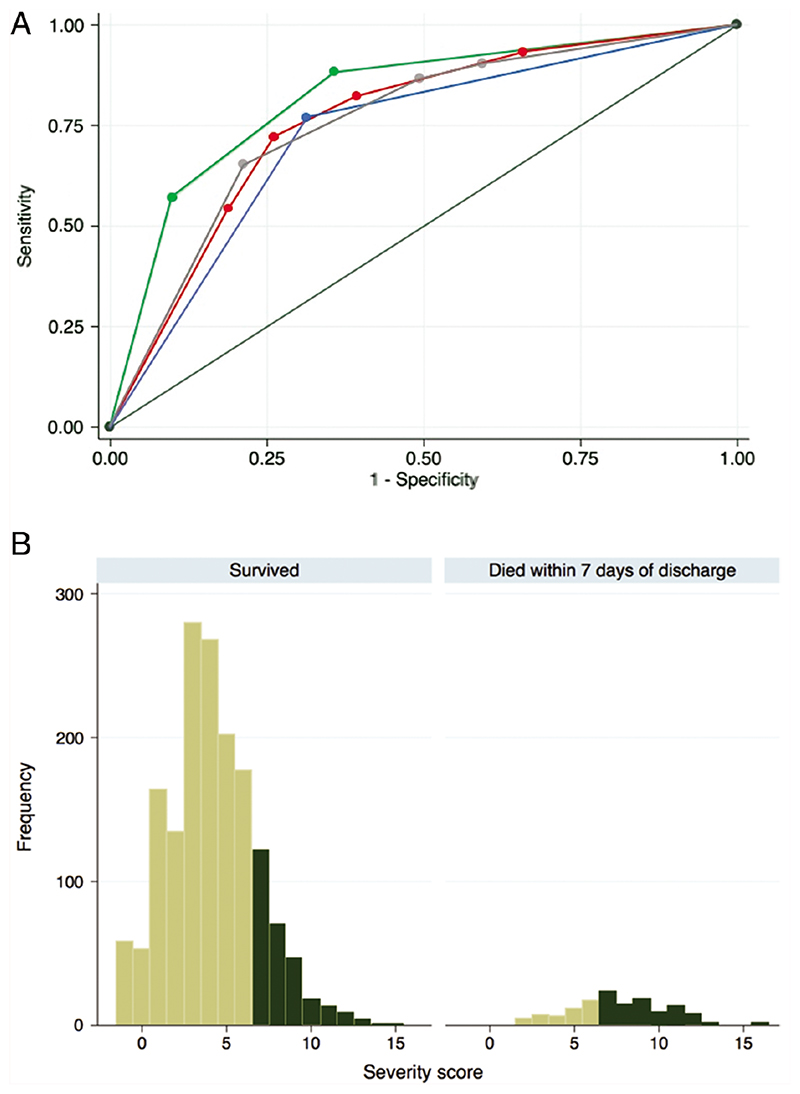
Predictive performance of the Pneumonia Etiology Research for Child Health (PERCH) 5-stratum severity score in the validation dataset compared to the World Health Organization (WHO) 2005 severity definitions, the number of WHO danger signs, and the Respiratory Index of Severity in Children (RISC) score. *A,* Receiver operating characteristic curves of each score/classification: WHO 2005 classification (the lowest, blue curve; area under the curve [AUC] = 0.73), RISC score (the second-lowest, red curve; AUC = 0.76), PERCH score (third-lowest, gray curve; AUC = 0.76), and danger signs classification (top, green curve; AUC = 0.82). *B,* Frequency plot of the total PERCH scores allocated to cases who died and cases who survived. The dark green columns indicate the children assigned to the highest- severity PERCH score stratum (stratum 5, with total scores between 7 and 17). The positive predictive value of the score indicates that 24% of children in the highest stratum (with a predicted probability of death of >0.2, dark green bars in the figure) actually died, ie, 24% of the dark green bars are in the right-hand panel. The specificity (79%) is the proportion of children who survived whose predicted probability of death was <0.2 (the children represented by light green in the left-hand panel, divided by the total number of children in the left-hand panel). The sensitivity (65%) is the proportion of children who died whose predicted probability of death was >0.2 (the children represented in dark green in the right-hand panel divided by the total in the right-hand panel). The negative predictive value indicates that 4.2% of children with a predicted probability of death <0.2 actually died.

In the validation dataset, the 5-stratum score was able to discriminate moderately well between children who died and those who survived (C statistic = 0.76; Figure 1). After bootstrapping to adjust for optimism, the C statistic remained at 0.76. No children allocated to stratum 1 died; 2.6% in strata 2 and 3 died; 7.1% in stratum 4 died; and 24% in stratum 5 died (Table 3). There was good calibration between the stratum-specific observed mortality and the mean predicted probabilities of mortality in the validation dataset, and they were similar to those in the development dataset (Supplementary Table 5). However, although the C statistic indicated that the score predicted the outcome across the population in the dataset moderately well, there was a large overlap in the total scores allocated to individuals who died and those who survived (Figure 1).

When the performance of the 5-stratum score was assessed by country, it performed moderately well in Zambia (C statistic = 0.72) and The Gambia (C statistic = 0.74), but performed less well in Kenya, Mali, and a combined dataset of SouthAfrica, Bangladesh, and Thailand (C statistics = 0.64–0.70). The 5-stratum score performed moderately well (C statistic = 0.74–0.76) in all age groups except 6- to 11-month-olds, where its discriminatory capacity dropped to 0.62 (Supplementary Table 6).

### Comparison of Available Scores

Observed mortality ranged from 17% in those classified with the WHO 2005 definition of very severe pneumonia, to 33% among children with ≥2 danger signs on presentation to hospital (Table 3).

The PERCH score (C statistic = 0.76), the RISC score (C statistic = 0.76), and the WHO 2005 classification (C statistic = 0.73) had similar abilities to discriminate between those who died and those who survived. The number of danger signs on presentation to hospital demonstrated the highest discriminatory capacity (C statistic = 0.82; Table 3, Figure 1). When assessing both discrimination and calibration, the danger signs were most predictive of mortality, followed by the PERCH 5-stratum score and RISC score; the WHO 2005 classification performed less well.

## Discussion

The model performed well and the 5-stratum severity score performed moderately well in predicting mortality when validated on a temporally discrete dataset. The ability of the PERCH 5-stratum score to discriminate between those children who died and those who survived was similar to the RISC score in HIV-negative children but had slightly better positive and negative predictive values. Importantly, the observed mortality in the lowest PERCH severity stratum was 0%—that is, the PERCH 5-stratum score performed well in identifying those who survived after admission to hospital. All PERCH cases were treated in hospital, so we cannot infer from this dataset which cases may have been successfully managed at home; this would have to be the subject of a new clinical trial.

When all PERCH cases were assigned to a PERCH severity stratum, the sites with the highest mortality correlated with those with the highest proportion of cases in the highest-severity stratum (Mali, Zambia; Supplementary Table 7).

In children presenting with WHO-defined severe or very severe pneumonia, observed cough was associated with survival, despite being a recognized sign of pneumonia. The absence of cough may indicate the presence of more severe signs of respiratory distress and/or severe weakness as the case defining feature, or the presence of sepsis, cerebral malaria, or meningitis, which are associated with higher mortality than pneumonia.

The utility of the available severity scores for clinicians in the management of childhood pneumonia is unclear, and the 2013 revision of the WHO severity classifications and recommendations for clinical care has been challenged [25]. A large study of 16 000 Kenyan children presenting to hospital with pneumonia found that 74% admitted to hospital under the WHO 2005 guidelines would have been classified as eligible for home treatment using the 2013 WHO guidelines; however, 3% of this group died after admission to hospital [17]. This finding is replicated in this analysis where 2.7% of children in the WHO 2005 severe category died after admission to hospital. The WHO 2005 classification of severe or very severe pneumonia performed least well in predicting mortality in the PERCH data.

The number of danger signs on presentation to hospital demonstrated the best ability to discriminate between those who survived and those who died when both ease of use and performance are considered. Few (1.5%) children who presented with no danger signs died, and the proportion of those children assigned to the highest stratum of severity who actually did die was the highest across the scores. These findings reinforce the utility of the WHO danger signs, including pulse oximetry, in the clinical management of pneumonia cases across diverse settings.

The methods used to develop the model and PERCH severity score are comparable to those used elsewhere [4] and adhere to standardized guidelines on the development and validation of multivariable prediction models for individual prognosis (Transparent Reporting of a Multivariable Prediction Model for Individual Prognosis or Diagnosis [TRIPOD] statement) [13]. We were able to assess a very large selection of variables, and our model identified similar factors to those identified in other pneumonia severity scores [4, 7, 8, 17]. The triage and treatment of pneumonia cases was standardized across countries to minimize the impact of indicator bias on mortality risk [15]. However, there were some differences; for example, the South African site administered oxygen to almost all cases on arrival, whereas other sites used pulse oximetry, if available, to guide oxygen administration. “Hypoxemia” in this analysis was defined using pulse oximetry or being on oxygen on arrival; the prevalence of hypoxemia at the South African site (78%) was therefore overestimated, which may have underestimated the contribution of “true” hypoxemia to mortality.

As the PERCH pneumonia severity model was derived from cases hospitalized with cough/difficulty breathing and either lower chest wall indrawing or danger signs, we cannot infer its performance among all cases of lower respiratory tract infection presenting to hospital, or among pneumonia cases treated in the community. The utility of the PERCH score needs to be validated with other, external datasets. Changes in etiology may influence severity, which may limit the utility of any score over time. Malaria tests were not relevant in all PERCH sites, and this limited the sample size with which to assess the importance of malaria in predicting mortality. In malaria-endemic sites (Kenya, The Gambia, Mali, and Zambia), 6.8% of the 44 HIVnegative children with a positive slide for malaria died within 7 days of discharge, compared to 9.8% of 989 with a negative slide (*P* = .5).

Risk factors for severe pneumonia and pneumonia mortality differ with HIV status. Developing a severity score for HIV-negative cases only (due to the limited number of HIVpositive cases, n = 251) restricts the utility of the PERCH score to clinical settings and studies where HIV status is known or where HIV prevalence is very low. WHO guidelines indicate that any HIV-positive child with suspected pneumonia should be hospitalized; therefore, the added utility of a severity score to triage HIV-positive cases is low. However, a study in Malawi found that 73% of cases of pneumonia presenting to hospital during 2011-2014 were missing information on HIV status [7]. Additionally, pulse oximetry is unavailable in many settings; only 50% of patients underwent oxygen saturation measurements in one observational study of routine care in Kenya [17]. Hypoxemia was strongly associated with mortality in this model (Supplementary Table 3) and others [4, 7], reinforcing the clinical utility of pulse oximetry.

## Conclusions

The 5-stratum severity score, derived from PERCH data on cases hospitalized with severe or very severe pneumonia in 7 LMICs, performed moderately well in predicting mortality in HIV-negative children. The lowest risk stratum predicted survival well, with no observed mortality. The PERCH score could be used to control for severity in analyses of geographic variations in pneumonia etiology. When compared to all available scores, the number of danger signs was most predictive of mortality and could be the most useful, of the currently available tools, to aid clinical management of pneumonia cases.

## Supplementary Material

Supplementary File

## Figures and Tables

**Table 1:** Factors Predictive of Mortality Among Human Immunodeficiency Virus-negative Children 1-59 Months of Age Presenting to Hospital With Severe or Very Severe Pneumonia: Multivariable Analyses

Characteristic	Died Within 7 d of Discharge				Unadjusted		Adjusted		
	No		Yes		OR^a^	(95% CI)	aOR	(95% CI)	*LRT P* Value^a^
All^b^	1682	(93.3)	120	(6.7)	...	...	...	...	
Site									
Kenya	372	(96.6)	13	(3.4)	1.06	(.48-2.35)	1.13	(.41-3.11)	
The Gambia	235	(95.5)	11	(4.5)	1.42	(.61-3.26)	2.13	(.73-6.27)	
Mali	213	(86.6)	33	(13.4)	4.69	(2.37-9.27)	2.20	(.84-5.75)	
Zambia	125	(74.0)	44	(26.0)	10.6	(5.45-20.8)	12.3	(5.13-29.7)	
South Africa	363	(96.8)	12	(3.2)	1	(ref)	1	(ref)	
Thailand	115	(97.5)	3	(2.5)	0.80	(.22-2.85)	0.77	(.09-6.69)	
Bangladesh	259	(98.5)	4	(1.5)	0.47	(.15-1.46)	1.00	(.25-3.99)	
Age, mo									
1-11	947	(91.9)	84	(8.1)	1.37	(.89-2.10)	2.20	(1.28-3.78)	.0031
12-59	735	(95.3)	36	(4.7)	1	(ref)	1	(ref)	
Sex									
Male	974	(95.0)	51	(5.0)	1	(ref)	1	(ref)	.0038
Female	708	(91.1)	69	(8.9)	1.76	(1.19-2.61)	1.99	(1.24-3.20)	
Unresponsiveness and/or deep breathing^c^									
Neither	1149	(95.2)	58	(4.8)	1	(ref)	1	(ref)	<.0001
Deep breathing, but alert	366	(95.8)	16	(4.2)	1.46	(.78-2.73)	1.18	(.58-2.39)	
Unresponsive but no deep breathing	123	(83.7)	24	(16.3)	4.61	(2.60-8.19)	3.12	(1.51-6.45)	
Unresponsive and deep breathing	36	(62.1)	22	(37.9)	19.4	(9.74-38.8)	14.6	(6.53-32.8)	
Cough (observed)									
No	498	(88)	68	(12)	1	(ref)	1	(ref)	.0032
Yes	1175	(95.8)	52	(4.2)	0.43	(.28-.65)	0.48	(.29-.78)	
Grunting (observed)									
No	1414	(95.8)	62	(4.2)	1	(ref)	1	(ref)	.0026
Yes	257	(81.6)	58	(18.4)	2.77	(1.67-4.58)	2.48	(1.37-4.48)	
Hypoxemia^d^									
No	1125	(95.7)	51	(4.3)	1	(ref)	1	(ref)	.0002
Yes	554	(88.9)	69	(11.1)	3.18	(2.08-4.86)	2.55	(1.54-4.22)	
Maximum duration of illness,^e^ d									
0-2	637	(96.5)	23	(3.5)	1	(ref)	1	(ref)	.0018
3-5	740	(93.4)	52	(6.6)	1.87	(1.11-3.15)	2.15	(1.16-3.99)	
>5	291	(87.4)	42	(12.6)	3.21	(1.84-5.63)	3.28	(1.66-6.46)	
Weight-for-height z-score									
Very low (< -3)	160	(82.5)	34	(175)	4.55	(2.75-755)	3.57	(2.03-6.31)	<.0001
Low (≥ -3 to < -2)	223	(91)	22	(9)	2.54	(1.47-4.41)	2.45	(1.32-4.52)	
Normal-high (≥ -2)	1258	(96)	52	(4)	1	(ref)	1	(ref)	

Data are presented as no. (%) unless otherwise indicated. Abbreviations: aOR, adjusted odds ratio; CI, confidence interval; d, days; LRT, likelihood ratio test; OR, odds ratio; ref, reference category.

a Children presenting to hospital with cough or difficulty breathing (observed or history of) and observed lower chest wall indrawing or at least 1 danger sign from the World Health Organization Pocketbook of Hospital Care for children were enrolled into the Pneumonia Etiology Research for Child Health (PERCH) study. The full set of univariable analyses, including information on missing data, is included in Supplementary Table 3; factors displayed in this table are only those that remained in the final multivariable model. All analyses controlled for site using “site” as a forced, indicator variable. *P* values were obtained from logistic regression LRT During backwards regression modeling, covariates were removed from the model if they did not significantly improve the fit of the model to the data (*P* > .05). When variables associated with the outcome in univariable analyses were added back into the model, they did not significantly increase the fit of the model to the data. When the model was run using random effects to control for clustering rather than logistic regression estimating fixed effects for each site, the coefficients were identical to those above and the allocated scores were the same.

b A total of 1719 of 1802 observations were used in the final model (95%); observations with missing data were significantly associated with higher mortality (*x* ^2^ *P* < .05), and their omission from the final model reduced the overall mortality from 6.7% to 6.2%.

c LRT *P* value for interaction = .018.

d Hypoxemia was defined as oxygen saturation <92% in all sites except Zambia and South Africa (the 2 sites situated at altitude >1000 m), where it was defined as <90%.

e Maximum reported duration of illness with fever, cough, difficulty breathing, or wheeze.

**Table 2:** Pneumonia Etiology Research for Child Health Score

Characteristic	Adjusted Log Coefficient	PERCH Score^a^
Age, mo		
1**-**11	0.79	+2
12-59	...	...
Sex		
Male	...	
Female	0.69	+1
Unresponsiveness and/or deep breathing		
Neither	...	...
Deep breathing, but alert	0.16	+0
Unresponsive but no deep breathing	1.14	+2
Unresponsive and deep breathing	2.68	+5
Cough (observed)		
No	...	...
Yes	0.74	-1
Grunting (observed)		
No	...	...
Yes	0.91	+2
Hypoxemia^b^		
No	...	...
Yes	0.94	+2
Maximum duration of illness^c^		
0 **-**2	...
3**-**5	0.77	+2
>5	1.19	+2
Weight-for-height z-score		
Very low (< -3)	1.27	+3
Low (≥ -3 to < -2)	0.90	+2
Normal-high (≥ -2)	...	...

Abbreviation: PERCH, Pneumonia Etiology Research for Child Health.

a The PERCH severity score was calculated by doubling the rounded log coefficients from the multivariable model (rounded to the nearest 0.5).

b Hypoxemia was defined as oxygen saturation <92% in all sites except Zambia and South Africa (the 2 sites situated at altitude), where it was defined as <90%.

c Maximum reported duration of illness with fever, cough, difficulty breathing, or wheeze.

**Table 3:** Calibration of Observed Versus Predicted Mortality in the Validation Dataset, by Score

Severity Strata	Observed Mortality,No. (%)		Crude OR (95% CI)	Mean Predicted Mortality, %	C Statistic (Adjusted for Optimism^a^)	PPV^b^	NPV^b^
PERCH score strata^c^							
-1 to 1	0/275	(0)	...	1.0	0.76 (0.76)	23.6%	95.8%
2	5/139	(3.6)	(ref)	1.9			
3-4	13/560	(2.3)	0.64 (.22-1.82)	2.8			
5-6	29/408	(7.1)	2.05 (.78-5.41)	6.8			
7-17	88/373	(23.6)	8.28 (3.28-20.9)	23.1			
Total	135/1755	...	...	...	...	...	...
WHO 2005 classification^01^							
Severe	31/1145	(2.7)	(ref)	2.7	0.73 (0.73)	0%	92.3%
Very severe	104/610	(170)	7.39 (4.88-11.2)	17.0			
Total	135/1755	...	...	...	...	...	...
South Africa RISC strata^e^							
-2 to 1	9/567	(1.6)	(ref)	1.6	0.76 (0.76)	19.4%	95.6%
2	15/440	(3.4)	2.19 (.95-5.05)	3.3			
3	13/227	(5.7)	3.77 (1.59-8.94)	6.9			
4	24/141	(17)	12.7 (5.76-28.1)	15			
5-8	73/376	(19.4)	14.9 (7.37-30.3)	19.6			
Total	134/1751	...	...	...	...	...	...
WHO danger signs, No.^f,g^							
0	14/916	(1.5)	(ref)	1.5	0.82 (0.82)	32.5%	96.1%
1	37/404	(9.2)	6.50 (3.47-12.2)	9.2			
≥2	67/206	(32.5)	31.1 (17.0-56.8)	32.5			
Total	118/1526	...	...	...	...	...	...

Abbreviations: CI, confidence interval; NPV, negative predictive value; OR, odds ratio; PERCH, Pneumonia Etiology Research for Child Health; PPV, positive predictive value; ref, reference category; RISC, Respiratory Index of Severity in Children; WHO, World Health Organization.

a Optimism is where the C statistic overestimates the score’s predictive ability due to overfitting of the model to the data, eg, when using a small dataset.

b As the predicted probabilities of death assigned to cases using the score alone were all <0.5 (range, 0.02-0.24), a cutoff of >0.2 was used to define a predicted death to calculate PPV and NPV.

c The decision to split the score into quintiles was set out in the statistical analysis plan and the split by frequency was computed using Stata software; groups are not exactly the same size given that we could not split groups of children assigned the same integer score. A total of 79 children were assigned a PERCH score of ≥10; observed mortality in this group was 42%. Of the 5 children who died with a PERCH score of 2, 3 were female, 2 were classified as very severe pneumonia, 4 were 12-59 months old, all were normal-high weight for height; 1 was lethargic, and 2 had <92% oxygen saturation at admission.

d Severe pneumonia is defined as cough/difficulty breathing and lower chest wall indrawing (LCWI); very severe pneumonia is cough/any difficulty breathing plus any one of the following danger signs: central cyanosis, inability to feed, vomiting everything, convulsions, lethargy, or severe respiratory distress (head nodding or grunting). Note that oxygen concentration was not used in the definition of very severe pneumonia.

e Characteristics included in the RISC score included oxygen saturation <90%, LCWI, low weight for age, refusal to feed, and wheeze. The validation dataset exhibited higher observed mortality than seen in the South African data from which the score was developed [1].

f WHO danger signs were defined as central cyanosis or oxygen saturation <90% on pulse oximetry, inability to drink/feed, vomiting everything, convulsions, lethargy/unresponsiveness or impaired consciousness, and severe respiratory distress (head nodding). The severity of chest wall indrawing was not noted in the dataset and therefore “severe chest wall indrawing” could not be included as a danger sign; all of those cases categorized with no danger signs displayed some LCWI (as it was a requirement for study eligibility [severe or very severe pneumonia as per WHO 2005 guidelines]). The sample size used to assess the performance of the PERCH, RISC, and WHO scores was larger than that possible to use for the danger signs score due to missing data on some of the danger signs. When restricted to only the population with a score based on danger signs, the C statistics were as follows: PERCH score, 0.79; WHO 2005 score, 0.75; RISC score, 0.81.

g Of the 14 children with no danger signs who died, 9 (65%) were female; 11 (79%) were aged 1-11 months; 10 (72%) were low/very low weight for height; 11 (79%) had illness duration for >3 days before presenting to hospital; none exhibited lethargy or unresponsiveness; only 3 were hypoxic (<92% oxygen); 9 (64%) had a cough. A total of 6 (43%) were in the highest PERCH risk stratum, and the remainder were equally distributed between the 3 other PERCH strata (not the lowest risk stratum with 0% mortality).
