# Supplementary material for: The Predictive Performance of a Pneumonia Severity Score in Human Immunodeficiency Virus-negative Children Presenting to Hospital in 7 Low- and Middle-income Countries
Source: Clin Infect Dis. Author manuscript; Available in PMC 2021 May 10. (PMC7610754; doi:10.1093/cid/ciz350)
Supplement: Supplementary File [file EMS123352-supplement-Supplementary_File.docx]

**Supplementary Table 1: World Health Organization classifications of pneumonia severity**

| Classification system | Severity strata | Symptoms | Treatment |
| --- | --- | --- | --- |
| WHO Pocket book of Hospital care for children – Guidelines for the management of common childhood illnesses 1^st^ ed. 2005[2] | Pneumonia | Fast breathing | Oral antibiotic for use at home |
|  | Severe pneumonia | Cough/ difficulty breathing (incl. fast breathing) plus at least one of lower chest wall indrawing, nasal flaring and/or grunting | Admit to hospital for injectable antibiotic and observation |
|  | Very Severe pneumonia | Difficulty breathing + (any danger sign^a^ or severe respiratory distress^b^) | Admit to hospital for injectable antibiotic +/- oxygen therapy |
| WHO Pocket book of Hospital care for children – Guidelines for the management of common childhood illnesses 2^nd^ ed. 2013[3] | Pneumonia | Fast breathing or chest in-drawing only | Oral antibiotic for use at home |
|  | Severe Pneumonia | Difficulty breathing + (any danger sign^a^ or severe respiratory distress^c^) | Admit to hospital for injectable antibiotic +/- oxygen therapy |

^a^ Danger signs include central cyanosis, inability to drink/feed, vomiting (everything), convulsions, lethargy/ unresponsiveness or impaired consciousness.

^b^ Severe respiratory distress in the 2005 edition: Very fast, labored breathing with use of auxiliary muscles for breathing (head nodding). Child appears to tire easily and is not able to feed because of respiratory distress.

^c^ Severe respiratory distress in 2013 edition: Severe chest indrawing, nasal flaring, grunting or head nodding in presence of very labored, fast, or gasping breathing. Child is unable to feed because of respiratory distress and tires easily.

**Supplementary Table 2: Existing clinical scores to define severity in hospitalized cases of lower respiratory tract infection**

| Indicator^a^ | RISC score[1] (South Africa) | | | | mRISC score[4] (Kenya) | | RISC-Malawi [5] (Malawi) |
| --- | --- | --- | --- | --- | --- | --- | --- |
|  | **Population:** Clinical trial participants <2 years of age who had received PCV9 and Hib vaccines but were hospitalized for LRTI during follow-up | | | | **Population:** Children <5 years old hospitalized with severe acute respiratory illness | | **Population:** children <5 years of age hospitalized with pneumonia |
|  | **HIV negative** | | **HIV positive** | | **All** | | **All** |
| Hypoxemia | <=90%: +3 | | <=90%: +2 | |  | | 90-92%: +2  <90%: +7 |
| Lower chest wall indrawing | +2 | | +1 | | +1 | |  |
| Low weight for age^b^ | very low: +2  low: +1 | |  | | Low/very low: +1 | |  |
| MUAC^c^ |  | |  | |  | | Low: +3  Very low: +7 |
| Refusal to feed | +1 | | +1 | | +1 | |  |
| Age |  | | 0-2 months: +2  3-12 months: +1 | |  | |  |
| Wheeze (observed) | -2 | | -1 | |  | | -2 |
| HIV status^d^ |  | | C: +2  A/B: +1  N: 0 | |  | |  |
| History of unconsciousness |  | |  | | +1 | |  |
| History of night sweats |  | |  | | -1 | |  |
| Not alert/awake |  | |  | | +2 | | unconscious: +8 |
| Positive malaria test |  | |  | | -1 | |  |
| Positive malaria test and chest wall indrawing |  | |  | | +1 | |  |
| Dehydration |  | |  | | +1 | |  |
| Female |  | |  | |  | | +1 |
| Maximum score | **Total 8** | | **Total 7** | | **Total 8** | | **Total 23** |
| Predicted mortality by score strata | Score of ≤1:  Score of 2:  Score of 3:  Score of 4:  Score of 5+: | <1%  1%  2%  4%  10-12% | Score of 1:  Score of 2:  Score of 3:  Score of 4:  Score of 5:  Score of 6+: | 2%  4%  7%  15%  27%  45% | Score of 1:  Score of 2:  Score of 3:  Score of 4:  Score of 5+: | 10%  20%  40%  56%  80% | NA |

# Abbreviations and footnotes: MUAC: mid-upper arm circumference; NA: not available.

# ^a^ If characteristic is present, the child is assigned the scores in the table, if characteristic is not present the child is assigned a score of 0. NA: not available. mRISC and RISC-Malawi were not assessed as a comparison in this publication given the different study populations and lack of information on history of unconsciousness, night sweats, and dehydration in the PERCH dataset.

^b^ Z-scores are categorized for weight for age, as follows: very low (<-3 SDs), Low (≥-3 - <-2 SDs), Normal-High (≥-2 SDs).

^c^ Low mid-upper arm circumference: 11.5-13.5; Very low MUAC: <11.5

^d^ HIV status: C is Severe, A/B is mild or moderate and N is not symptomatic

**Supplementary Table 3. Univariable analyses of all candidate predictive variables against outcome of “death (in hospital or within 7 days of discharge)” among HIV negative children 1-59 months of age in the development dataset, controlling for country site**

| Characteristic | | Died in hospital or within 7 days of discharge (%) | | Total | Crude OR [95%CI] | LRT p-value^a^ |
| --- | --- | --- | --- | --- | --- | --- |
|  |  | **No** | **Yes** |  |  |  |
| ALL^b^ | | 1682 (93.3) | 120 (6.7) | 1802 |  |  |
| Site^a^ | Kenya | 372 (96.6) | 13 (3.4) | 385 | 1.06 (0.48-2.35) | <0.0001 |
|  | Gambia | 235 (95.5) | 11 (4.5) | 246 | 1.42 (0.61-3.26) |  |
|  | Mali | 213 (86.6) | 33 (13.4) | 246 | 4.69 (2.37-9.27) |  |
|  | Zambia | 125 (74.0) | 44 (26.0) | 169 | 10.6 (5.45-20.8) |  |
|  | South Africa | 363 (96.8) | 12 (3.2) | 375 | 1 |  |
|  | Thailand | 115 (97.5) | 3 (2.5) | 118 | 0.80 (0.22-2.85) |  |
|  | Bangladesh | 259 (98.5) | 4 (1.5) | 263 | 0.47 (0.15-1.46) |  |
| Demographics | | | | | | |
| Age - median (iqr) | Months | 10 (4-19) | 6 (3-12) | 1802 | 0.98 (0.97-1.00) | 0.1076 |
| Age (4) | 1-5 months | 573 (90.7) | 59 (9.3) | 632 | 1.57 (0.84-2.94) | 0.3698  (p-trend: 0.9117) |
|  | 6-11 months | 374 (93.7) | 25 (6.3) | 399 | 1.22 (0.61-2.44) |  |
|  | 12-23 months | 439 (95.2) | 22 (4.8) | 461 | 1.08 (0.53-2.19) |  |
|  | 24-59 months | 296 (95.5) | 14 (4.5) | 310 | 1 |  |
| Age (2) | 1-11 months | 947 (91.9) | 84 (8.1) | 1031 | 1.37 (0.89-2.10) | 0.1449 |
|  | 12-59 months | 735 (95.3) | 36 (4.7) | 771 | 1 |  |
| Sex | Male | 974 (95.0) | 51 (5.0) | 1025 | 1 | 0.0043 |
|  | Female | 708 (91.1) | 69 (8.9) | 777 | 1.76 (1.19-2.61) |  |
| Signs on clinical examination | | | | | | |
| Lower chest wall in-drawing | No | 139 (89.7) | 16 (10.3) | 155 | 1 | 0.0008 |
|  | Yes | 1543 (93.7) | 104 (6.3) | 1647 | 0.31 (0.16-0.59) |  |
| Head nodding | No | 1449 (93.8) | 95 (6.2) | 1544 | 1 | 0.1196 |
|  | Yes | 232 (90.3) | 25 (9.7) | 257 | 1.50 (0.91-2.45) |  |
|  | . | 1 |  |  |  |  |
| Central Cyanosis | No | 1649 (93.7) | 110 (6.3) | 1759 | 1 | 0.0159 |
|  | Yes | 30 (75) | 10 (25) | 40 | 2.86 (1.28-6.37) |  |
|  | Missing | 2 |  |  |  |  |
| Unable to feed | No | 1584 (94.6) | 90 (5.4) | 1674 | 1 | <0.0001 |
|  | Yes | 97 (76.4) | 30 (23.6) | 127 | 4.63 (2.80-7.67) |  |
| Vomiting | No | 1630 (93.4) | 116 (6.6) | 1746 | 1 | 0.4680 |
|  | Yes | 51 (92.7) | 4 (7.3) | 55 | 1.53 (0.51- 4.58) |  |
|  | Missing | 1 |  |  |  |  |
| Unresponsiveness or impaired consciousness (4) | Alert/awake | 1518 (95.4) | 73 (4.6) | 1591 | 1 | <0.0001 |
|  | Responds to voice | 116 (85.3) | 20 (14.7) | 136 | 6.06 (3.21-11.4) |  |
|  | Responds to pain | 35 (67.3) | 17 (32.7) | 52 | 6.28 (3.15-12.5) |  |
|  | Unresponsive | 9 (50) | 9 (50) | 18 | 17.6 (5.96-51.8) |  |
| Any unresponsiveness | No | 1520 (95.4) | 74 (4.6) | 1594 | 1 | <0.0001 |
|  | Yes | 160 (77.7) | 46 (22.3) | 206 | 7.10 (4.48-11.3) |  |
|  | Missing | 2 |  |  |  |  |
| Convulsions | No | 1589 (93.6) | 109 (6.4) | 1698 | 1 | 0.0758 |
|  | Yes | 92 (89.3) | 11 (10.7) | 103 | 1.97 (0.97- 3.98) |  |
|  | Missing | 1 |  |  |  |  |
| O2 saturation (3) | <60% | 7 (44) | 9 (56) | 16 | 9.29 (3.13-27.5) | <0.0001 |
|  | 60-91% or “on oxygen at admission” | 548 (90.1) | 60 (9.9) | 608 | 2.89 (1.87-4.47) |  |
|  | >=92 | 1126 (95.7) | 51 (4.3) | 1177 | 1 |  |
| Hypoxaemia^c^ | No | 1125 (95.7) | 51 (4.3) | 1176 | 1 | <0.0001 |
|  | Yes | 554 (88.9) | 69 (11.1) | 623 | 3.18 (2.08-4.86) |  |
|  | Missing | 2 |  |  |  |  |
| Elevated Resp. rate^d^ | No | 320 (92.5) | 26 (7.5) | 346 | 1 | 0.1861 |
|  | Yes | 1356 (93.6) | 93 (6.4) | 1449 | 0.70 (0.43-1.14) |  |
|  | Missing | 6 | 1 |  |  |  |
| Elevated heart rate^d^ | No | 842 (94.5) | 49 (5.5) | 891 | 1 | 0.6466 |
|  | Yes | 838 (92.2) | 71 (7.8) | 909 | 1.10 (0.74-1.64) |  |
|  | Missing | 2 |  |  |  |  |
| Fever (≥38°C on examination) | No | 336 (94.9) | 18 (5.1) | 354 | 1 | 0.5252 |
|  | Yes | 1346 (93.0) | 102 (7.0) | 1448 | 1.19 (0.69-2.07) |  |
| Cough (observed) | No | 498 (88.0) | 68 (12.0) | 566 | 1 | <0.0001 |
|  | Yes | 1175 (95.8) | 52 (4.2) | 1227 | 0.43 (0.28-0.65) |  |
|  | Missing | 7 |  |  |  |  |
| Wheeze on auscultation | No | 1052 (90.9) | 105 (9.1) | 1157 | 1 | 0.0032 |
|  | Yes | 615 (98.1) | 12 (1.9) | 627 | 0.37 (0.18-0.77) |  |
|  | Missing | 13 | 3 |  |  |  |
| Skin Turgor | Normal | 1610 (94.3) | 97 (5.7) | 1707 | 1 | <0.0001 |
|  | Reduced | 65 (73.9) | 23 (26.1) | 88 | 4.48 (2.54-7.90) |  |
|  | Missing | 4 |  |  |  |  |
| Capillary refill time | <2 seconds | 721 (98.6) | 10 (1.4) | 731 | 1 | 0.0143 |
|  | 2-3 seconds | 623 (90.8) | 63 (9.2) | 686 | 2.61 (1.17-5.84) |  |
|  | >3 seconds | 31 (77.5) | 9 (22.5) | 40 | 5.42 (1.70-17.3) |  |
|  | Missing | 307 | 38 |  |  |  |
| Crackling/ crepitations on auscultation | No | 628 (93.2) | 46 (6.8) | 674 | 1 | 0.3094 |
|  | Yes | 1043 (93.5) | 72 (6.5) | 1115 | 1.24 (0.82-1.87) |  |
|  | Missing | 10 | 2 |  |  |  |
| Grunting | No | 1414 (95.8) | 62 (4.2) | 1476 | 1 | 0.0001 |
|  | Yes | 257 (81.6) | 58 (18.4) | 315 | 2.77 (1.67-4.58) |  |
|  | Missing | 8 |  |  |  |  |
| Stridor | No | 1628 (93.2) | 119 (6.8) | 1747 | 1 | 0.2246 |
|  | Yes | 47 (97.9) | 1 (2.1) | 48 | 0.35 (0.05-2.60) |  |
|  | Missing | 6 |  |  |  |  |
| Nasal flaring | No | 711 (94.3) | 43 (5.7) | 754 | 1 | 0.6190 |
|  | Yes | 966 (92.6) | 77 (7.4) | 1043 | 1.12 (0.71-1.76) |  |
|  | Missing | 4 |  |  |  |  |
| Deep breathing | No | 1274 (94.0) | 82 (6.0) | 1356 | 1 | <0.0001 |
|  | Yes | 402 (91.4) | 38 (8.6) | 440 | 2.69 (1.70-4.27) |  |
|  | Missing | 5 |  |  |  |  |
| At least one danger sign | No | 959 (97.1) | 29 (2.9) | 988 | 1 | <0.0001 |
|  | Yes | 723 (88.8) | 91 (11.2) | 814 | 4.80 (3.02-7.63) |  |
| Medical history | | | | | | |
| History of difficulty breathing | No | 142 (97.9) | 3 (2.1) | 145 | 1 | 0.0802 |
|  | Yes | 1539 (92.9) | 117 (7.1) | 1656 | 2.52 (0.77-8.23) |  |
|  | Missing | 1 |  |  |  |  |
| History of cough | No | 54 (71.1) | 22 (28.9) | 76 | 1 | <0.0001 |
|  | Yes | 1627 (94.3) | 98 (5.7) | 1725 | 0.23 (0.12-0.41) |  |
|  | Missing |  |  |  |  |  |
| History of unable to feed | No | 1504 (94.6) | 86 (5.4) | 1590 | 1 | <0.0001 |
|  | Yes | 176 (83.8) | 34 (16.2) | 210 | 3.54 (2.21-5.67) |  |
|  | Missing | 1 |  |  |  |  |
| History of ear discharge | No | 1669 (93.3) | 119 (6.7) | 1788 | 1 | 0.9527 |
|  | Yes | 12 (92.3) | 1 (7.7) | 13 | 0.94 (0.12-7.61) |  |
|  |  |  |  |  |  |  |
| History of diarrhea | No | 1452 (94.8) | 80 (5.2) | 1532 | 1 | 0.0002 |
|  | Yes | 228 (85.1) | 40 (14.9) | 268 | 2.37 (1.53-3.66) |  |
|  | Missing | 2 |  |  |  |  |
| Maximum duration of illness with fever, cough, difficulty breathing or wheeze | 0-2 days | 637 (96.5) | 23 (3.5) | 660 | 1 | 0.0002  (p-trend 0.85) |
|  | 3-5 days | 740 (93.4) | 52 (6.6) | 792 | 1.87 (1.11-3.15) |  |
|  | >5 days | 291 (87.4) | 42 (12.6) | 333 | 3.21 (1.84-5.63) |  |
|  | Missing | 14 | 3 |  |  |  |
| Prior diagnosis for wheeze | No | 1503 (93.1) | 112 (6.9) | 1615 | 1 | 0.2314 |
|  | Yes | 158 (97.5) | 4 (2.5) | 162 | 0.55 (0.20-1.57) |  |
|  | Missing | 18 | 4 |  |  |  |
| HIV exposure^e^ | Unexposed | 1388 (94.2) | 86 (5.8) | 1474 | 1 | 0.2496 |
|  | Exposed | 182 (87.5) | 26 (12.5) | 208 | 1.64 (0.91-2.96) |  |
|  | Unknown | 112 (93.3) | 8 (6.7) | 120 | 0.94 (0.43-2.03) |  |
| Medication/ vaccination history | | | | | | |
| Prior admission to hospital since birth | No | 1180 (92.9) | 90 (7.1) | 1370 | 1 | 0.9319 |
|  | Yes | 494 (95.4) | 24 (4.6) | 518 | 1.02 (0.62-1.68) |  |
|  | Missing | 8 | 6 |  |  |  |
| Any medication | No | 670 (94.1) | 42 (5.9) | 712 | 1 | 0.7219 |
|  | Yes | 989 (92.8) | 77 (7.2) | 1066 | 0.92 (0.60-1.42) |  |
|  | Missing | 22 | 1 |  |  |  |
| DTP vaccination status appropriate for age | None | 254 (93.0) | 19 (7.0) | 273 | 0.89 (0.51-1.54) | 0.3370 |
|  | Partial | 187 (95.9) | 8 (4.1) | 195 | 0.58 (0.27-1.26) |  |
|  | Full | 1189 (93.3) | 85 (6.7) | 1274 | 1 |  |
|  | Missing | 52 | 8 |  |  |  |
| Hib vaccination status appropriate for age | None | 358 (94.7) | 20 (5.3) | 378 | 0.80 (0.45-1.41) | 0.3624 |
|  | Partial | 153 (96.2) | 6 (3.8) | 159 | 0.58 (0.24-1.40) |  |
|  | Full | 1119 (92.9) | 86 (7.1) | 1205 | 1 |  |
|  | Missing | 52 | 8 |  |  |  |
| PCV vaccination status appropriate for age | None | 755 (91.7) | 68 (8.3) | 823 | 0.90 (0.49-1.65) | 0.7504 |
|  | Partial | 191 (96.5) | 7 (3.5) | 198 | 0.7 (0.32-1.70) |  |
|  | Full | 690 (94.7) | 39 (5.3) | 729 | 1 |  |
|  | Missing | 46 | 6 |  |  |  |
| Birth history/ growth | | | | | | |
| Premature | No | 1386 (93.5) | 96 (6.5) | 1482 | 1 | 0.2455 |
|  | Yes | 235 (93.6) | 16 (6.4) | 251 | 1.43 (0.79-2.58) |  |
|  | Missing | 61 | 8 |  |  |  |
| Birthweight | Normal-high (>2490g) | 1282 (93.8) | 85 (6.2) | 1367 | 1 | 0.5284 |
|  | Low (≤ 2490g) | 385 (93.2) | 28 (6.8) | 413 | 1.16 (0.73-1.86) |  |
|  | Missing | 15 | 7 |  |  |  |
| Premature or small at birth | No | 1210 (93.8) | 80 (6.2) | 1290 | 1 | 0.4205 |
|  | Yes | 464 (93.5) | 33 (6.6) | 497 | 1.20 (0.77-1.87) |  |
|  | Missing | 8 | 7 |  |  |  |
| Height for age | Very low (<-3 SDs) | 263 (88.0) | 36 (12.0) | 299 | 1.72 (1.04-2.85) | 0.1151 |
|  | Low (≥-3 - <-2 SDs) | 261 (93.6) | 18 (6.5) | 279 | 1.28 (0.72-2.28) |  |
|  | Normal-high (≥-2 SDs) | 1129 (95.1) | 58 (4.9) | 1187 | 1 |  |
|  | Missing | 29 | 8 |  |  |  |
| Weight for age | Very low (<-3 SDs) | 249 (84.4) | 46 (15.6) | 295 | 4.43 (2.78-7.04) | <0.0001 |
|  | Low (≥-3 - <-2 SDs) | 335 (91.8) | 30 (8.2) | 365 | 2.39 (1.44-3.97) |  |
|  | Normal-high (≥-2 SDs) | 1094 (96.2) | 43 (3.8) | 1137 | 1 |  |
|  | Missing | 4 | 1 |  |  |  |
| Weight for height | Very low (<-3 SDs) | 160 (82.5) | 34 (17.5) | 194 | 4.55 (2.75-7.55) | <0.0001 |
|  | Low (≥-3 - <-2 SDs) | 223 (91.0) | 22 (9.0) | 245 | 2.54 (1.47-4.41) |  |
|  | Normal-high (≥-2 SDs) | 1258 (96.0) | 52 (4.0) | 1310 | 1 |  |
|  | Missing | 41 | 12 |  |  |  |
| BMI for age | Very low (<-3 SDs) | 173 (82.4) | 37 (17.6) | 210 | 4.21 (2.58-6.86) | <0.0001 |
|  | Low (≥-3 - <-2 SDs) | 221 (92.9) | 17 (7.1) | 238 | 1.87 (1.03-3.38) |  |
|  | Normal-high (≥-2 SDs) | 1258 (95.7) | 57 (4.3) | 1315 | 1 |  |
|  | Missing | 30 | 9 |  |  |  |
| Diagnostics | | | | | | |
| Chest radiograph(3) | Abnormal (consolidation/ infiltrate) | 798 (94.4) | 47 (5.6) | 845 | 1.95 (1.13-3.36) | 0.0130 |
|  | Normal | 658 (97.1) | 20(2.9) | 678 | 1 |  |
|  | uninterpretable | 162 (91.0) | 16 (9.0) | 178 | 2.56 (1.25-5.23) |  |
|  | Missing | 64 | 37 |  |  |  |
| Leukocytosis | No | 890 (93.4) | 63 (6.6) | 953 | 1 | 0.4175 |
|  | Yes | 725 (93.3) | 52 (6.7) | 777 | 1.18 (0.79-1.76) |  |
|  | Missing | 67 | 5 |  |  |  |
| Anemia^f^ (3) | None  (Hb >= 9.3 g/dl) | 1205 (94.7) | 67 (5.3) | 1272 | 1 | 0.0015 |
|  | Non-severe  (Hb 6-9.2 g/dl) | 372 (91.2) | 36 (8.8) | 408 | 1.44 (0.91-2.26) |  |
|  | Moderate- severe anemia  (Hb <6 g/dl) | 37(77.1) | 11(22.9) | 48 | 4.60 (2.09-10.1) |  |
|  | Missing | 68 | 6 |  |  |  |
| Anemia (2) | None  (Hb >= 9.3 g/dl) | 1205 (94.7) | 67 (5.3) | 1272 | 1 | 0.0147 |
|  | Anemic | 409 (89.7) | 47 (10.3) | 456 | 1.70 (1.11-2.60) |  |
|  | Missing | 68 | 6 |  |  |  |
| C reactive protein | 0-40 mg/L (~ 80% viral infections) | 1059 (94.5) | 62 (5.5) | 1121 | 1 | 0.1095 |
|  | >40 mg/L (~ 80% bacterial infections) | 411 (90.1) | 45 (9.9) | 456 | 1.42 (0.93-2.17) |  |
|  | Missing | 212 | 13 |  |  |  |
| Malaria test result^g^ | Negative | 1629 (93.4) | 116 (6.7) | 1745 | 1 | 0.8673 |
|  | Positive | 41 (93.2) | 3 (6.8) | 44 | 1.11 (0.32-3.81) |  |
|  | Missing | 12 | 1 |  |  |  |
| Environmental factors | | | | | | |
| Crowding – number sleeping in the same room as child | 1-2 people | 250 (93.6) | 17 (6.4) | 267 | 1 | 0.9449 |
|  | 3-4 people | 1116 (93.1) | 83 (6.9) | 1199 | 0.91 (0.51-1.60) |  |
|  | 5 or more (up to 17) | 307 (93.9) | 20 (6.1) | 327 | 0.91 (0.44-1.85) |  |
|  | Missing | 9 | 0 |  |  |  |
| Solid fuel | No | 562 (92.9) | 43 (7.1) | 605 | 1 | 0.0798 |
|  | Yes | 1112 (93.4) | 76 (6.4) | 1188 | 0.59 (0.33-1.07) |  |
|  | Missing | 8 | 1 |  |  |  |
| Exposure to smoke | No | 1131 (92.6) | 91 (7.4) | 1222 | 1 | 0.23271 |
|  | Yes | 544 (95.3) | 27 (4.7) | 571 | 0.79 (0.49-1.28) |  |
|  | Missing | 7 | 2 |  |  |  |

^a^ Children presenting to hospital with cough or difficulty breathing (observed or history of) and observed LCWI or WHO danger sign were enrolled onto the PERCH study. P-values obtained from logistic regression likelihood ratio test, across all cases presenting to hospital using ‘site’ as a forced, indicator variable. Variables were assessed in the multivariable logistic regression model where univariate analyses suggest association p<0.2.

^b^ 192 missing observations of outcome among cases who had no vital status data at 30 days’ post discharge

^c^ Hypoxemia was defined as oxygen saturation <90% in Zambia and South Africa and <92% at all other sites, or the child was already on oxygen at admission.

^d^ Raised respiratory rate at admission defined as: < 2 months of age: ≥60 breaths/min, 2-11 months: ≥50 breaths/min, 12-59 months: ≥40 breaths/min. Elevated heart rate on admission was defined as: if 1-11 months of age: >160 bpm, 12-35 months: >150 bpm, 36-59 months: >140 bpm.

^e^ HIV-exposure remained unassociated with mortality when analysis was restricted to high prevalence sites (South Africa and Zambia) where 12% of those exposed died, compared to 9% of those unexposed (p=0.09)

^f^ Anemia was classified as per the WHO guidelines for the management of common childhood illnesses (2013 ed.).

^g^ Malaria tests were not conducted in South Africa, Bangladesh or Thailand where prevalence is thought to be <1%; missing results for these sites were imputed as negative for the purposes of these univariate analyses.

**Supplementary Table 4.** **Characteristics of the populations used to develop and validate the PERCH severity score**

| Characteristic | Development dataset  19^th^ Aug 2011 – 7^th^ Nov 2012 | | | | | | Validation dataset  7^th^ Nov 2012 – 26^th^ Jan 2014 | |  |  |  |
| --- | --- | --- | --- | --- | --- | --- | --- | --- | --- | --- | --- |
|  | **n** | | | **%** | | | **n** | **%** |  |  |  |
| Deaths in hospital or within 7 days of discharge among HIV negative children |  | 120/1802 | | 6.7 | | | 146/1819 | 8.0 |  |  |  |
| Site^a^ | **1802** | | | **-** | | | **1819** | **-** |  |  |  |
| Kenya | 385 | | | 21.4 | | | 226 | 12.4 |  |  |  |
| Gambia | 246 | | | 13.7 | | | 379 | 20.8 |  |  |  |
| Mali | 246 | | | 13.7 | | | 381 | 20.9 |  |  |  |
| Zambia | 169 | | | 9.4 | | | 130 | 7.1 |  |  |  |
| South Africa | 375 | | | 20.8 | | | 351 | 19.3 |  |  |  |
| Thailand | 118 | | | 6.5 | | | 100 | 5.5 |  |  |  |
| Bangladesh | 263 | | | 14.6 | | | 252 | 13.9 |  |  |  |
| Median age (months; IQR) | 9 (4-19) | | | - | | | 7 (3-15) | - |  |  |  |
| Age | **1802** | | | **-** | | | **1819** | **-** |  |  |  |
| 1-5 months | 632 | | | 35.1 | | | 802 | 44.1 |  |  |  |
| 6-11 months | 399 | | | 22.1 | | | 419 | 23.0 |  |  |  |
| 12-23 months | 461 | | | 25.6 | | | 388 | 21.3 |  |  |  |
| 24-59 months | 310 | | | 17.2 | | | 210 | 11.5 |  |  |  |
| Sex | **1802** | | | **-** | | | **1819** |  |  | | |
| Male | 1025 | | | 56.9 | | | 1076 | 59.2 |  | | |
| Female | 777 | | | 43.1 | | | 743 | 40.8 |  | | |
| At least one danger sign | **1802** | | |  | | | **1819** |  |  | | |
| No | 988 | | | 54.8 | | | 966 | 53.1 |  | | |
| Yes | 814 | | | 45.2 | | | 853 | 46.9 |  | | |
| Maximum duration of illness^b^ | **1785** | |  | | **1816** | | |  |  | |  |
| 0-2 days | 660 | | | 37.0 | | | 610 | 33.6 |  | | |
| 3-5 days | 792 | | | 44.4 | | | 864 | 47.6 |  | | |
| >5 days | 333 | | | 18.7 | | | 342 | 18.8 |  | | |
| DTP vaccination status appropriate for age | **1742** | | |  | | | **1760** |  |  | | |
| None | 273 | | | 15.7 | | | 327 | 18.9 |  | | |
| Partial | 195 | | | 11.2 | | | 220 | 12.5 |  | | |
| Full | 1274 | | | 73.1 | | | 1213 | 68.9 |  | | |
| Hib vaccination status appropriate for age | **1742** | | |  | | | **1761** |  |  | | |
| None | 378 | | | 21.7 | | | 412 | 23.4 |  | | |
| Partial | 159 | | | 9.1 | | | 168 | 9.5 |  | | |
| Full | 1205 | | | 69.2 | | | 1181 | 67.1 |  | | |
| PCV vaccination status appropriate for age | **1750** | | |  | | **1766** | |  | |  |  |
| None | 823 | | | 47 | | 759 | | 43.0 |  |  |  |
| Partial | 198 | | | 11.3 | | 193 | | 10.9 |  |  |  |
| Full | 729 | | | 41.7 | | 814 | | 46.1 |  |  |  |
| Birthweight | **1780** | | |  | | **1799** | |  |  |  |  |
| Normal (>2490g) | 1367 | | | 76.8 | | 1478 | | 82.2 |  |  |  |
| Low (≤ 2490g) | 413 | | | 23.2 | | 321 | | 17.8 |  |  |  |

Abbreviations and footnotes: IQR: Inter-quartile range.

^a^ The total number of observations available and column percentages are presented for each characteristic

^b^ Maximum reported duration of illness with fever, cough, difficulty breathing or wheeze

**Supplementary Table 5. PERCH score performance, on the development dataset, omitting site**

| Score strata | Score category | Observed mortality^a^ | | Crude OR  95% CI | Mean predicted mortality % |
| --- | --- | --- | --- | --- | --- |
|  |  | **n** | **%** |  |  |
| 1 | -1 to 1 | 1/254 | 0.4 | 1 | 0.4 |
| 2 | 2 | 7/383 | 1.8 | 4.71 (0.58-38.5) | 1.1 |
| 3 | 3 to 4 | 3/262 | 1.2 | 2.93 (0.30-28.4) | 2.0 |
| 4 | 5 to 6 | 14/377 | 3.7 | 9.76 (1.28-74.7) | 4.3 |
| 5 | 7 to 17 | 78/363 | 21.5 | 69.2 (9.56-501) | 20.9 |

^a^ mortality in hospital or within 7 days of discharge; 78 children were assigned a score of 10 or higher with an observed mortality of 47% but the definition of a stratum using such low numbers risks poor validation later

**Supplementary Table 6. Score performance in the validation dataset; by site and by age group**

| Sub-group | Score strata | Observed mortality in hospital or within 7 days of discharge | | Mean predicted mortality | c-statistic | c-statistic (adjusted for optimism) |
| --- | --- | --- | --- | --- | --- | --- |
|  |  | **n** | **%** | % |  |  |
| Site |  |  |  |  |  |  |
| Kenya |  |  |  |  | 0.64 | 0.64 |
|  | -1 to 1 | 0/24 | 0 | 1.0 |  |  |
|  | 2 | 0/18 | 0 | 2.0 |  |  |
|  | 3 to 4 | 3/64 | 4.7 | 3.5 |  |  |
|  | 5 to 6 | 8/52 | 15.4 | 9.1 |  |  |
|  | 7 to 17 | 5/57 | 8.8 | 24.8 |  |  |
| The Gambia | |  |  |  | 0.74 | 0.72 |
|  | -1 to 1 | 0/62 | 0 | 0.9 |  |  |
|  | 2 | 1/48 | 2.1 | 1.6 |  |  |
|  | 3 to 4 | 0/155 | 0 | 2.4 |  |  |
|  | 5 to 6 | 4/78 | 5.1 | 5.5 |  |  |
|  | 7 to 17 | 7/31 | 22.6 | 20.6 |  |  |
| Mali |  |  |  |  | 0.70 | 0.70 |
|  | -1 to 1 | 0/13 | 0 | 1.1 |  |  |
|  | 2 | 1/7 | 14.3 | 2.0 |  |  |
|  | 3 to 4 | 5/63 | 7.9 | 3.3 |  |  |
|  | 5 to 6 | 5/105 | 4.8 | 7.5 |  |  |
|  | 7 to 17 | 56/192 | 29.2 | 24.4 |  |  |
| Zambia |  |  |  |  | 0.75 | 0.74 |
|  | -1 to 1 | 0/7 | 0 | 0.7 |  |  |
|  | 2 | 3/9 | 33.3 | 1.9 |  |  |
|  | 3 to 4 | 4/43 | 9.3 | 2.4 |  |  |
|  | 5 to 6 | 6/33 | 18.2 | 6.8 |  |  |
|  | 7 to 17 | 14/26 | 53.9 | 28.0 |  |  |
| South Africa, Thailand, Bangladesh^a^ | | |  |  | 0.77 | 0.68 |
|  | -1 to 1 | 0/169 | 0 | 1.1 |  |  |
|  | 2 | 0/57 | 0 | 2.1 |  |  |
|  | 3 to 4 | 1/235 | 0.4 | 2.8 |  |  |
|  | 5 to 6 | 6/140 | 4.3 | 6.1 |  |  |
|  | 7 to 17 | 6/67 | 9.0 | 17.0 |  |  |
| Age group | |  |  |  |  |  |
| Age 1-5 months | |  |  |  | 0.77 | 0.76 |
|  | -1 to 1 | 0/39 | 0 | 0.7 |  |  |
|  | 2 | 0/30 | 0 | 1.2 |  |  |
|  | 3 to 4 | 3/275 | 1.1 | 2.2 |  |  |
|  | 5 to 6 | 15/226 | 6.6 | 5.5 |  |  |
|  | 7 to 17 | 40/199 | 20.1 | 18.4 |  |  |
| Age 6-11 months | |  |  |  | 0.74 | 0.62 |
|  | -1 to 1 | 0/19 | 0 | 0.7 |  |  |
|  | 2 | 1/22 | 4.5 | 1.2 |  |  |
|  | 3 to 4 | 5/146 | 3.4 | 2.2 |  |  |
|  | 5 to 6 | 4/109 | 3.7 | 5.7 |  |  |
|  | 7 to 17 | 25/112 | 22.3 | 24.6 |  |  |
| Age 12-23 months | |  |  |  | 0.75 | 0.74 |
|  | -1 to 1 | 0/124 | 0 | 1.1 |  |  |
|  | 2 | 4/57 | 7.0 | 2.2 |  |  |
|  | 3 to 4 | 4/100 | 4.0 | 4.5 |  |  |
|  | 5 to 6 | 7/51 | 13.7 | 12.3 |  |  |
|  | 7 to 17 | 14/42 | 33.3 | 34.5 |  |  |
| Age 24-59 months | |  |  |  | 0.82 | 0.76 |
|  | -1 to 1 | 0/93 | 0 | 1.1 |  |  |
|  | 2 | 0/30 | 0 | 2.5 |  |  |
|  | 3 to 4 | 1/39 | 2.6 | 4.6 |  |  |
|  | 5 to 6 | 3/22 | 13.6 | 13.1 |  |  |
|  | 7 to 17 | 9/20 | 45.0 | 38.1 |  |  |

^a^ Data were combined to enable performance statistics to be estimated due to the low number of events.

**Supplementary Table 7: Application of the score to describe the heterogeneity in the case-mix presenting to hospital across the whole PERCH dataset of cases.**

| Sub-group | Score strata | | Proportion of cases allocated to the severity strata | Observed mortality in hospital or within 7 days of discharge | | |  |
| --- | --- | --- | --- | --- | --- | --- | --- |
|  |  |  |  | **n** | **%** | |  |
| All PERCH cases |  | |  |  |  | |  |
|  | -1 to 1 | | 18% | 1/574 | 0.2 | |  |
|  | 2 | | 10% | 7/299 | 2.3 | |  |
|  | 3 to 4 | | 31% | 20/974 | 2.1 | |  |
|  | 5 to 6 | | 23% | 42/709 | 5.9 | |  |
|  | 7 to 17 | | 18% | 133/572 | 23.3 | |  |
| Total |  | |  | 203/3128 | 6.5 | |  |
| Site |  | |  |  |  | |  |
| Kenya |  | |  |  |  | |  |
|  | -1 to 1 | | 17% | 1/90 | 1.1 | |  |
|  | 2 | | 12% | 0/63 | 0 | |  |
|  | 3 to 4 | | 30% | 3/157 | 1.9 | |  |
|  | 5 to 6 | | 22% | 9/116 | 7.8 | |  |
|  | 7 to 17 | | 19% | 10/103 | 9.7 | |  |
| Total |  | |  | 23/529 | 4.3 | |  |
| The Gambia | | |  |  |  | |  |
|  | -1 to 1 | | 22% | 0/111 | 0 | |  |
|  | 2 | | 13% | 1/68 | 1.5 | |  |
|  | 3 to 4 | | 36% | 1/187 | 0.5 | |  |
|  | 5 to 6 | | 19% | 3/99 | 3.0 | |  |
|  | 7 to 17 | | 10% | 15/50 | 30.0 | |  |
| Total |  | |  | 20/515 | 3.9 | |  |
| Mali |  | |  |  |  | |  |
|  | -1 to 1 | | 3% | 0/14 | 0 | |  |
|  | 2 | | 2% | 2/7 | 28.6 | |  |
|  | 3 to 4 | | 18% | 4/78 | 5.1 | |  |
|  | 5 to 6 | | 28% | 5/121 | 4.1 | |  |
|  | 7 to 17 | | 49% | 58/212 | 27.4 | |  |
|  |  | |  | 69/432 | 16.0 | |  |
| Zambia |  | |  |  |  | |  |
|  | -1 to 1 | | 8% | 0/22 | 0 | |  |
|  | 2 | | 7% | 3/20 | 15.0 | |  |
|  | 3 to 4 | | 31% | 9/83 | 10.8 | |  |
|  | 5 to 6 | | 30% | 16/80 | 20 | |  |
|  | 7 to 17 | | 24% | 36/65 | 55.4 | |  |
| Total |  | |  | 64/270 | 23.7 | |  |
| South Africa | |  | | | |  | |
|  | -1 to 1 | | 12% | 0/80 | 0 | |  |
|  | 2 | | 7% | 0/45 | 0 | |  |
|  | 3 to 4 | | 30% | 1/199 | 0.5 | |  |
|  | 5 to 6 | | 35% | 7/233 | 3.0 | |  |
|  | 7 to 17 | | 16% | 9/106 | 8.5 | |  |
| Total |  | |  | 17/663 | 2.6 | |  |
| Thailand |  | |  |  |  | |  |
|  | -1 to 1 | | 40% | 0/82 | 0 | |  |
|  | 2 | | 13% | 0/27 | 0 | |  |
|  | 3 to 4 | | 29% | 1/59 | 1.7 | |  |
|  | 5 to 6 | | 9% | 1/19 | 5.3 | |  |
|  | 7 to 17 | | 9% | 3/18 | 16.7 | |  |
| Total |  | |  | 5/205 | 2.4 | |  |
| Bangladesh |  | |  |  |  | |  |
|  | -1 to 1 | | 34% | 0/175 | 0 | |  |
|  | 2 | | 13% | 1/69 | 1.4 | |  |
|  | 3 to 4 | | 41% | 1/211 | 0.5 | |  |
|  | 5 to 6 | | 8% | 1/41 | 2.4 | |  |
|  | 7 to 17 | | 4% | 2/18 | 11.1 | |  |
|  |  | |  | 5/514 | 1.0 | |  |
| Chest X-ray positive only^a^ | | | |  |  | |  |
|  | -1 to 1 | | 17% | 0/236 | 0 | |  |
|  | 2 | | 9% | 2/133 | 1.5 | |  |
|  | 3 to 4 | | 30% | 10/429 | 2.3 | |  |
|  | 5 to 6 | | 25% | 18/347 | 5.2 | |  |
|  | 7 to 17 | | 19% | 54/270 | 20.0 | |  |
|  |  | |  | 84/1415 | 5.9 | |  |

^a^ Participants with abnormal Chest X-ray findings (consolidation or infiltrate)

**Supplementary Table 8: Characteristics of the HIV negative cases missing outcome (mortality) data**

| Characteristic | Participants with mortality data | | | Participants missing mortality data | |
| --- | --- | --- | --- | --- | --- |
|  | **n** | | **%** | **n** | **%** |
| All |  | 3621/3962 | 91.4 | 341/3962 | 8.6 |
| Site^a^ |  | |  |  |  |
| Kenya | 611 | | 16.9 | 4 | 1.2 |
| Gambia | 625 | | 17.3 | 6 | 1.8 |
| Mali | 627 | | 17.3 | 22 | 6.5 |
| Zambia | 299 | | 8.3 | 215 | 63.1 |
| South Africa | 726 | | 20.1 | 79 | 23.2 |
| Thailand | 218 | | 6.0 | 5 | 1.5 |
| Bangladesh | 515 | | 14.2 | 10 | 2.9 |
| Median age (months; IQR) | 8 (3-17) | |  | 5 (2-11) |  |
| Age |  | |  |  | **-** |
| 1-5 months | 1434 | | 39.6 | 178 | 52.2 |
| 6-11 months | 818 | | 22.6 | 83 | 24.3 |
| 12-23 months | 849 | | 23.5 | 54 | 15.8 |
| 24-59 months | 520 | | 14.4 | 26 | 7.6 |
| Sex |  | |  |  |  |
| Male | 2101 | | 58.0 | 191 | 56.0 |
| Female | 1520 | | 42.0 | 150 | 44.0 |
| At least one danger sign^b^ |  | |  |  |  |
| No | 1954 | | 54.0 | 177 | 51.9 |
| Yes | 1667 | | 46.0 | 164 | 48.1 |
| Maximum duration of illness^c^ |  |  |  | |  |
| 0-2 days | 1270 | | 35.3 | 117 | 34.6 |
| 3-5 days | 1656 | | 46.0 | 171 | 50.6 |
| >5 days | 675 | | 18.7 | 50 | 14.8 |
| Birthweight |  | |  |  |  |
| Normal (>2490g) | 2845 | | 79.5 | 279 | 83.8 |
| Low (≤ 2490g) | 734 | | 20.5 | 54 | 16.2 |
| Hypoxemia |  | |  |  |  |
| >=92% | 2336 | | 64.7 | 198 | 58.2 |
| <92% | 1277 | | 34.3 | 142 | 41.8 |

Abbreviations and footnotes: IQR: Inter-quartile range.

^a^ The total number of observations available and column percentages are presented for each characteristic. Fieldworkers returned to homesteads several times during follow-up in order to attempt to obtain outcome data. Participants missing outcome data were excluded from the model given the small percentage of participants this represented.

^b^ At least one danger sign of: central cyanosis, inability to drink/feed, vomiting (everything), convulsions, lethargy/ unresponsiveness or impaired consciousness

^c^ Maximum reported duration of illness with fever, cough, difficulty breathing or wheeze

**Supplementary Figure 1. Internal validation of the predictive performance of the 5-stratum severity score (using the development dataset)**

| 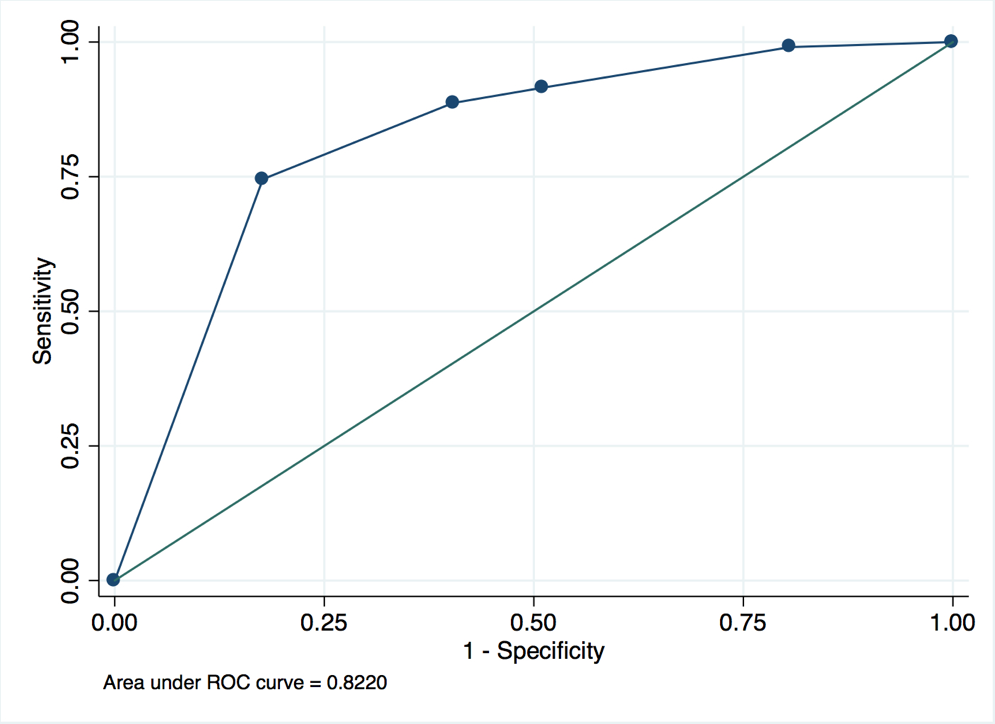 | | | | |
| --- | --- | --- | --- | --- |
| 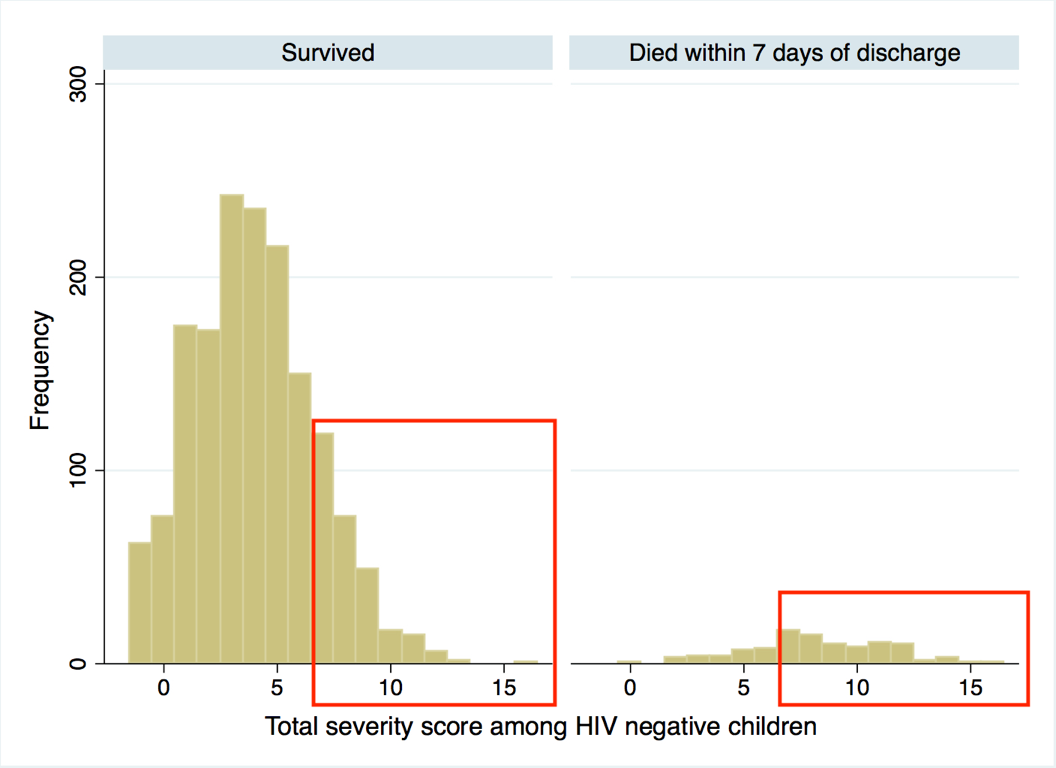 | | | | |
| **Pr. Cut-off^a^** | **Sensitivity** | **Specificity** | **Pos. predictive value** | **Neg. predictive value** |
| >0.2 | 74.5% | 82.3% | 21.7% | 98.0% |

^a^ The predicted probability of death used as a cut-off to define a ‘predicted death’. A severity score in the highest stratum (stratum 5, equivalent to a total score of 7-17) corresponds to a predicted probability of death of on average, >20% (mean 21%). The specificity (82.3%) is the proportion of children who survived whose predicted probability of death was <20% (left panel). The sensitivity (75%) is the proportion of children who died whose predicted probability of death was >20% (right hand panel). The positive predictive value indicates that 22% of children with a predicted probability of death of >0.2, died. Red boxes indicate the children assigned to the highest severity score stratum (7-17).

1. Reed C, Madhi SA, Klugman KP, et al. Development of the Respiratory Index of Severity in Children (RISC) score among young children with respiratory infections in South Africa. PloS one **2012**; 7(1): e27793.

2. World Health Organization. Pocket book of hospital care for children: guidelines for the management of common childhood illnesses – 1st ed. Geneva, **2005**.

3. World Health Organization. Pocket book of hospital care for children: guidelines for the management of common childhood illnesses – 2nd ed. Geneva, **2013**.

4. Emukule GO, McMorrow M, Ulloa C, et al. Predicting Mortality among Hospitalized Children with Respiratory Illness in Western Kenya, 2009–2012. PloS one **2014**; 9(3): e92968.

5. Hooli S, Colbourn T, Lufesi N, et al. Predicting Hospitalised Paediatric Pneumonia Mortality Risk: An External Validation of RISC and mRISC, and Local Tool Development (RISC-Malawi) from Malawi. PloS one **2016**; 11(12): e0168126.
